# TARGETED TRANSCRANIAL DIRECT CURRENT STIMULATION IMPROVES DUAL-TASK WALKING PERFORMANCE IN OLDER ADULTS

**DOI:** 10.1093/geroni/igz038.2923

**Published:** 2019-11-08

**Authors:** Brad Manor, Junhong Zhou, On-Yee Lo, Racheli Katz, Marina Brozgol, Pablo Cornejo Thumm, Giulio Ruffini, Jeffrey M Hausdorff

**Affiliations:** 1 Hinda and Arthur Marcus Institute for Aging Research, Harvard Medical School, Roslindale, Massachusetts, United States; 2 Tel Aviv Sourasky Medical Center, Tel Aviv, Israel; 3 Neuroelectrics Corporation, Cambridge, Massachusetts, United States; 4 Center for the study of Movement, Cognition and Mobility, Neurological Institute, Tel Aviv Sourasky Medical Center, Israel, Tel Aviv, Israel

## Abstract

In older adults, the ability to walk while engaged in an unrelated cognitive task (i.e., dual tasking) depends upon activation of both motor and cognitive brain networks. Noninvasive transcranial direct current stimulation (tDCS) can facilitate the excitability of specific brain regions and their connected neural networks. In this multi-site, randomized controlled within-subject cross-over study, we tested the effects of single, 20-minute sessions of tDCS targeting 1) the primary motor cortex (M1), 2) the left dorsolateral prefrontal cortex (dlPFC, a primary region subserving cognitive function), 3) both M1 and left dlPFC, or 4) neither region (sham). Forty-eight older adults free of overt illness or disease (mean±SD age=75±6 years, 35 women) completed four study visits at least 72 hours apart, during which dual task gait was assessed before and after tDCS administration. Stimulation was delivered using the Starstim™ system (Neuroelectrics Corp) and the same array of six gel electrodes to ensure double-blinding. Participants were successfully blinded to tDCS condition and reported no unexpected tDCS side effects. Repeated-measures ANOVAs adjusted for age and sex revealed that the dual task cost to gait speed was smaller (i.e., better and closer to zero) following tDCS that targeted both M1 and the left dlPFC, as well as the left dlPFC alone, compared to all other time points (condition-time interaction: F=3.0, p=0.04). The dual task costs following these two types of stimulation were similar. These results suggest that noninvasive facilitation of cognitive-motor brain network excitability leads to acute improvement in dual task performance in older adults.

